# Repurposing of paroxetine and fluoxetine for their antibacterial effects against clinical *Pseudomonas aeruginosa* isolates in Egypt

**DOI:** 10.3934/microbiol.2025007

**Published:** 2025-02-05

**Authors:** Kholoud Baraka, Rania Abozahra, Eman Khalaf, Mahmoud Elsayed Bennaya, Sarah M. Abdelhamid

**Affiliations:** 1 Microbiology and Immunology Department, Faculty of Pharmacy, Damanhour University, Damanhour, Egypt; 2 Microbiology Department, Faculty of Pharmacy, Menoufia University, Menoufia, Egypt

**Keywords:** fluoxetine, Egypt, PCR, paroxetine, *Pseudomonas*

## Abstract

**Background:**

Drug repositioning has emerged as a promising strategy for assessing its antimicrobial efficacy in treating infectious diseases.

**Methods:**

Seventy-five samples were collected and investigated for the presence of *Pseudomonas aeruginosa*. Antibiotic resistance, hemolytic activity, twitching motility, and biofilm formation were assessed. *lasI* and *lasR* genes were detected using conventional PCR. Minimum inhibitory concentrations of paroxetine, fluoxetine, and levofloxacin were determined by broth micro-dilution. The fractional inhibitory concentration index was calculated to assess the interaction between fluoxetine/levofloxacin and paroxetine/levofloxacin combinations. Half the MIC values of the drugs were selected for inhibitory effect assessment for virulence factors. Antibacterial and healing effects of fluoxetine were investigated on 30 male albino rats using a digital camera, bacterial count, and histological examination.

**Results:**

Our 25 *P. aeruginosa* isolates were highly drug-resistant. 80%, 92%, and 80% of isolates were positive for twitching motility, hemolysis, and biofilm formation, respectively. 92% of isolates were positive for *lasI* gene and 96% for *lasR* gene. MICs of fluoxetine and paroxetine ranged from 32 to 512 µg/mL and MICs of levofloxacin ranged from 1 to 256 µg/mL. A synergistic outcome was observed in both combinations. Biofilm formation, twitching motility, and hemolysis were inhibited by paroxetine and fluoxetine in the majority of isolates. Fluoxetine/levofloxacin and paroxetine/levofloxacin combinations inhibited twitching motility, hemolysis, and biofilm formation in all isolates. Enhanced wound healing was observed in rats treated with fluoxetine and levofloxacin, with the fluoxetine/levofloxacin combination group demonstrating the most significant wound-healing effect. Bacterial count decreased in rats treated with levofloxacin, fluoxetine, and the levofloxacin/fluoxetine combination. Histological examination revealed higher wound healing in the levofloxacin-treated group than the fluoxetine group, and the combination treatment group displayed the fastest rate of wound healing.

**Conclusions:**

Paroxetine and fluoxetine showed considerable antibacterial inhibitory effects against multi-drug resistant *P. aeruginosa* isolates. Fluoxetine showed significant improvement in anti-inflammatory effects and wound healing. To the best of our knowledge, this is the first Egyptian study to investigate the repurposing of paroxetine and fluoxetine as antibacterial agents. Further studies are needed to investigate their applicability as antibacterial agents as single agents or in combination with other antibiotics.

## 1. Introduction

Bacterial resistance is a global emergency and public health concern according to the World Health Organization (WHO) and the Centers for Disease Control and Prevention (CDC). It was estimated that resistance to traditional therapies would result in the death of seven million people by the year 2050 [1]–[4]. Horizontal transfer of resistance genes, environmental accumulation, and misuse and abuse of antibiotics are common causes of infections by multidrug-resistant (MDR) microorganisms [1],[5].

Unfortunately, the development of new antibiotics by pharmaceutical companies remains limited [6]. Bacterial infections are now being treated with a variety of ‘non-antibiotic’ medications [7]. Furthermore, medications used in combination with antimicrobials are presently employed as alternative therapies, reducing the potential of resistance [8],[9]. Therefore, drug repositioning has emerged as a promising strategy for assessing their antimicrobial efficacy in treating infectious diseases [10]. In other words, drug repositioning is the process of turning an existing medication toward a new use besides its original intended medical indication [11]–[13]. The term *repositioning* is also used to describe the repurposing, recycling, rediscovery, or reuse of drugs and refers to the process of identifying new therapeutic applications for existing medications [14],[15]. These drugs are distinguished by having the capacity to act on several targets [16]. In recent years, there has been more interest in drug repositioning as a potential approach. Lengthy licensing processes and challenges in drug development have hindered the pursuit of novel medications. As a result, researchers are now searching for novel, efficient treatment approaches to existing drugs [2],[6].

*In vitro* studies showed that antidepressants had an antimicrobial effect against MDR bacterial infections because of their ability to function as efflux pump inhibitors [17]. Traditionally, obsessive-compulsive disorder (OCD) and depression have been treated with selective serotonin reuptake inhibitors (SSRIs), such as fluoxetine and paroxetine [18]. Nonetheless, a number of studies emphasized the potential secondary actions of these drugs, including antioxidant, antibacterial, antifungal, anti-inflammatory, and anti-apoptotic activities [10],[19].

Slow-healing wounds are not only costly but represent a major public health concern for many patients. Wounds that heal slowly or never at all are mostly responsible for high morbidity and mortality. An uncontrolled, self-sustaining inflammatory response is one of the main factors behind the failure of chronic wound healing. The process of wound healing is intricate and involves the interplay of several cell types, cytokines, and other mediators. The four stages of wound healing are hemostasis, inflammation, proliferation, and tissue remodeling or disintegration, all being intricately related to one another [20]. These phases and their biophysiological functions must occur in the correct sequence, at the right time, and with appropriate intensity for the necessary duration [21]. Approximately 3–6 million Americans suffer from non-healing wounds, with 85% of cases occurring in individuals aged 65 years or older [22]. Furthermore, infections caused by microorganisms can also result in wounds that fail to heal. SSRI medications have gained widespread acceptance in the last years for their ability to control inflammation, exhibit antibacterial action, and help in the healing of wounds [23].

This study aimed to investigate the antibacterial effect of paroxetine and fluoxetine against antibiotic-resistant clinical isolates of *P. aeruginosa*, both individually and in combination with the antibacterial drug levofloxacin, to explore their potential for repositioning. Besides, it aimed to investigate the anti-inflammatory and healing effects of fluoxetine for clinical wounds.

## 2. Materials and methods

### 2.1. Sample collection, isolation, and identification

Seventy-five different samples were collected from patients at the National Liver Institute and Menoufia University Hospital, Menoufia, Egypt from March 2021 to January 2022. Urine, burns, ascetic fluid, and blood samples were collected. For the detection of *P. aeruginosa*, samples were cultured on MacConkey agar plates. Gram staining was performed on non-lactose fermenting colonies. Several biochemical tests were conducted, including oxidase, triple sugar iron agar, indole, methyl red, Voges-Proskauer, and citrate test [24]. Isolates were aerobically cultured on cetrimide agar plates. *P. aeruginosa* isolates were identified at the species level using the automated VITEK 2 system (bioMérieux, l'Etoile, France).

### 2.2. Antibiotic susceptibility testing

Antibiotic resistance was determined by the standard disc agar diffusion technique according to Bauer et al. [25]. Ten commercially available antibiotic discs (Oxoid®, UK), representing different antibiotic classes, were used to assess resistance to piperacillin (100 µg), piperacillin/tazobactam (110 µg), ceftazidime (30 µg), aztreonam (30 µg), cefepime (30 µg), gentamicin (10 µg), levofloxacin (5 µg), gatifloxacin (5 µg), meropenem (10 µg), and imipenem (10 µg). The results were interpreted according to CLSI 2020 guidelines [26].

### 2.3. Hemolytic activity of *P. aeruginosa* isolates

Five microliters of each *P. aeruginosa* strain were streaked onto nutrient agar plates supplemented with 5% sheep erythrocytes and incubated at 37 °C for 24 h. The colonies were then examined for β-hemolysis [27].

### 2.4. Twitching motility of *P. aeruginosa* isolates

Each *P. aeruginosa* strain was stabbed with a sterile toothpick into the bottom of a 1% Luria-Bertani (LB) agar plate into the agar layer without reaching the base of the plate and incubated at 37 °C for 24 h. The diffuse bacterial zone between the agar layer and the bottom of the polystyrene Petri dish served as a measure of twitching motility [28].

### 2.5. Biofilm assay of *P. aeruginosa* isolates

Biofilm formation in all *P. aeruginosa* isolates was evaluated using two methods: the qualitative Congo red agar (CRA) method and a semi-quantitative method.

#### 2.5.1. Biofilm assay using the qualitative CRA method

An aqueous solution containing 0.8 g of Congo red stain (Research Lab Fine Chem Industries, India) per 200 mL of distilled water was prepared and autoclaved. 50 g of sucrose and 37 g of brain heart infusion agar were dissolved in 800 mL of distilled water and then autoclaved. After cooling the agar to 55 °C, 200 mL of Congo red stain was added. *P. aeruginosa* isolates were inoculated into the prepared media and incubated aerobically for 24 h at 37 °C. Strong biofilm production is indicated by black colonies with a dry, crystalline quality. Red colonies that occasionally had black patches in their centers were considered non-biofilm producers [29].

#### 2.5.2. Biofilm assay using the semi-quantitative method

Briefly, a sterile 96-well microtiter well plate was inoculated with 125 µL of diluted overnight nutrient broth cultures of isolates and then incubated for 24 h at 37 °C. After three rounds of washing with 300 µL of distilled water to remove excess bacteria, each well was allowed to air dry at room temperature in an inverted position. Then, 125 µL of 0.1% crystal violet was added to the wells and left for 10–15 min at room temperature. Wells were rinsed three times with distilled water to remove the excess stain. 200 µL of 95% ethanol was added to the wells to elute crystal violet from biofilms, and the absorbance of the solubilized dye was measured at 620 nm using a microtiter plate reader (STATFAX2100, Fisher Bioblock Scientific, France). The assays were performed according to O'Toole's methodology [30] with minor modifications.

### 2.6. Molecular detection of quorum-sensing genes

Molecular detection of the quorum-sensing genes of *P. aeruginosa* isolates was performed in the Microbiology and Immunology Department, Faculty of Pharmacy, Damanhour University.

#### 2.6.1. DNA extraction

DNA was extracted directly by using the boiling method with some modifications. Briefly, 4–5 pure bacterial colonies of each isolate cultivated on nutrient agar plates were suspended in 500 µL of sterile water in a sterile Eppendorf tube and heated at 95 °C for 10 min. Bacterial suspensions were cooled on ice for 5 min and then centrifuged at 14,000 rpm for 5 min. Supernatants were transferred to sterile Eppendorf tubes and stored at −20 °C [31].

#### 2.6.2. Conventional PCR technique

DNA extracts were tested for the presence of *lasI* and *lasR* quorum sensing genes by using a thermal cycler (SimpliAmp Applied Biosystems, ThermoFisher®, USA) and DreamTaq PCR Master Mix (ThermoFisher®). Two pairs of primers were used (Table 1). PCR amplicons were resolved on a 1.5% agarose gel stained with ethidium bromide and visualized using ultraviolet illumination. The following cycling parameters were used: initial denaturation at 95 °C for 3 min, followed by 35 cycles of denaturation at 95 °C for 30 s, annealing at 50 °C for 30 s, and extension at 72 °C for 1 min, with a final extension at 72 °C for 10 min.

**Table 1.** Specific primers used for the molecular detection of quorum-sensing genes.

Gene	Primer sequence	Amplicon size (bp)	Reference
*lasR*	*lasR*–F: ATGGCCTTGGTTGACGGTT *lasR*–R: GCAAGATCAGAGAGTAATAAGACCCA	725 bp	[32]
*lasI*	*lasI*–F: ATGATCGTACAAATTGGTCGGC *lasI*–R: GTCATGAAACCGCCAGTCG	605 bp	

### 2.7. Detection of the effects of antidepressant drugs on QS-dependent virulence factors in *P. aeruginosa* isolates

#### 2.7.1. Determination of the antidepressant drugs (paroxetine and fluoxetine) minimum inhibitory concentration (MIC)

The MIC of paroxetine and fluoxetine was determined against *P. aeruginosa* isolates by the broth microdilution method [33]. Briefly, the antidepressant drugs were 2-fold serially diluted with sterile nutrient broth (NB). Then, 100 µL of these dilutions were distributed in the wells of a 96-well polystyrene microtiter plate. Each well received 10 µL of overnight diluted broth cultures of *P. aeruginosa* isolates to reach a final inoculum concentration of approximately 10^6^ CFU/mL in wells. To determine the MIC values, the plate was checked visually for microbial growth after incubation at 37 °C for 24 h. Inoculated wells without any drugs and uninoculated wells containing antidepressant drugs were included in the experiment as controls. The MIC value of the antidepressant drugs for each tested isolate was defined as the lowest concentration required to inhibit the visible growth of the microorganism. Half the MIC of the antidepressant drugs was selected for the assessment of their anti-QS and anti-biofilm activities in these isolates.

#### 2.7.2. Determination of the fractional inhibitory concentration index (FICI)

The FICI was calculated to analyze the interaction between the fluoxetine/levofloxacin and paroxetine/levofloxacin combinations, classified as synergistic (FICI ≤ 0.5), non-interactive (0.5 < FICI ≤ 4.0), or antagonistic (FICI > 4.0), using the following equations [34],[35]:

$$\begin{array}{c} \text{Drug A FICI}=\text{Drug A MIC in combination}÷\text{Drug A MIC alone}\\ \text{Drug B FICI}=\text{Drug B MIC in combination}÷\text{Drug B MIC alone}\\ \text{FICI}=\text{Drug A FICI}+\text{Drug B FICI}. \end{array}$$

#### 2.7.3. Biofilm inhibition assay

The effect of 1/2 MIC of antidepressant drugs and the fluoxetine/levofloxacin and paroxetine/levofloxacin combinations (1/2 MIC of each drug) on biofilm formation was determined using the microtiter plate method. Briefly, overnight NB broth cultures of tested isolates were diluted 1:100 into fresh NB, and then 200 µL of the freshly inoculated medium was dispensed into the wells of a 96-well polystyrene microtiter plate. Each strain was represented by six successive wells in the microtiter plate: three untreated wells containing bacterial suspension without antidepressant drugs as controls and three wells containing bacterial suspension with 1/2 MIC of antidepressant drugs. The plate was incubated at 37 °C for 24 h without agitation, and the assay was completed as previously mentioned in 5.2.

#### 2.7.4. Twitching motility inhibition assay

The effect of 1/2 MIC of antidepressant drugs and the fluoxetine/levofloxacin and paroxetine/levofloxacin combinations (1/2 MIC of each drug) on twitching motility was determined as follows. Tested *P. aeruginosa* isolates were stabbed using sterile toothpicks into LB agar plates supplemented with 1/2 MIC of antidepressant drugs. Plates were then incubated overnight at 37 °C and then left for 1–2 days at room temperature (<25 °C).

#### 2.7.5. Hemolytic activity inhibition assay

The effect of 1/2 MIC of antidepressant drugs and the fluoxetine/levofloxacin and paroxetine/levofloxacin combinations (1/2 MIC of each drug) on hemolytic activity was determined as follows. 5 µL of bacterial suspension of each *P. aeruginosa* strain was streaked on agar plates supplemented with 5% sheep erythrocytes and 1/2 MIC of antidepressant drugs followed by an overnight incubation at 37 °C. Bacterial colonies were then checked for signs of β-hemolysis.

### 2.8. Conventional scanning electron microscope (SEM)

Conventional SEM is one of the most suitable technologies for biofilm visualization, as it provides an appropriate description of biofilm morphology through high magnification and high-resolution pictures. Half MIC of paroxetine, fluoxetine, and levofloxacin were used. To visualize 3D images of cells, samples were coated with heavy metals such as gold. Electrons released from the metal coating of the sample are caught by SEM for image production [36]. The detailed methodology was as follows: Tiny cover slides (18 × 18 mm) were placed in six wells with bacterial suspensions of *P. aeruginosa* isolate (P1) added to three wells as controls. Half the MICs of paroxetine (64 µg/mL), fluoxetine (256 µg/mL), and levofloxacin (0.5 µg/mL) were individually added to the P1 suspensions in the remaining three wells. Wells were then incubated for 24 h. Then, glutaraldehyde, 2% paraformaldehyde, and 0.1 M sodium phosphate buffer (pH 7.4) were added, followed by three 15-min washes with 0.1 M sodium phosphate buffer and 0.1 M sucrose. Wells were dehydrated twice for 15 min in 50% ethanol after being post-fixed for 90 min in 2% sodium phosphate-buffered osmium tetroxide (pH 7.4) and rinsed three times for 15 min each. The contrast was developed overnight at 4 °C with 70% acetone, 0.5% uranyl acetate, and 1% phosphotungstic acid. Finally, the samples were washed twice for 15 min in 80% ethanol, twice in 90% ethanol, twice in 96% ethanol, and three times for 20 min in 100% ethanol. The specimens (on upper covers) were examined on a JEOL JSM-6510 LV SEM (JEOL, Japan) at the EM Unit of Mansoura University in Egypt after being coated with gold-palladium membranes. The microscope was run at 30 kV.

### 2.9. Determination of the antibacterial effect and healing of fluoxetine in vivo

#### 2.9.1. Preparation of bacterial cultures

Bacterial suspensions of *P. aeruginosa* in Müller-Hinton broth were incubated for 24 h at 37 °C and subsequently adjusted to a concentration of 5 × 10^9^ CFU/mL.

#### 2.9.2. Preparation of animals

Thirty male albino rats (150–200 g) were used in this study. The experiment was carried out in accordance with the guidelines established by the National Research Council (NRC) for the care and use of laboratory animals. Animals were supplied with a standard diet and ad libitum water. Prior to the experiment, all rats were given at least a week to acclimate to the laboratory environment, and all procedures were carried out in sterile conditions. Rats were divided into five groups, each group containing six rats. Group I was left without any wounds or infection receiving only carboxymethyl cellulose (CMC) as negative controls. Group II had wounds and was infected with the selected most virulent *P. aeruginosa* isolates. This group did not receive any medication and received only CMC to act as positive controls. Group III had wounds, was infected with the selected most virulent *P. aeruginosa* isolates, and was treated orally with 0.7 mg of fluoxetine. Group IV had wounds, was infected with the selected most virulent *P. aeruginosa* isolates, and was treated orally with half a dose of fluoxetine (0.35 mg) added to the levofloxacin antibiotic (8 mg). Group V had wounds, was infected with *P. aeruginosa* isolates, and was treated orally only with 16 mg of levofloxacin antibiotic.

#### 2.9.3. Experimental animal protocol

Rats were given intra-peritoneal injections of thiopental (60 mg/kg). Rats' dorsal skin was shaved, and each animal got a mark applied to its back measuring roughly 5.0 cm^3^. After that, a sterile biopsy punch needle with a diameter of 5 mm was used to produce a consistent wound (No. 5, Kai Industries Co., Ltd., Seki City, Japan). Then, 1 mL of bacterial suspension (5 × 10^9^ CFU/mL) was inoculated into the wound to produce a bacterial infection by gently massaging the entire shaved region with a cotton-tipped swab until no visible fluid remained. Swabs were collected from the affected area before and after the infection to compare the number of bacterial colonies and to confirm that all groups had similar infections. Infected areas were covered with sterile bandages and maintained immobile for 24 h before the start of the treatment. Infection was induced below occlusive covers. Signs of inflammation, including redness, fever, and an increased bacterial count, were observed to confirm the infection. Treated rats were given a single oral daily dose of fluoxetine (3 mg per rat) for 7 days within 24 h of the infection discovery.

#### 2.9.4. Wound healing measurement

Wounds in each group were photographed with a digital camera at various intervals (0, 1, 3, 5, and 7 days).

#### 2.9.5. Bacterial count determination

Bacterial count in each group was performed according to Yu et al. [37] with minor modifications. Wound exudates were collected using sterile cotton swabs infiltrated with sterile normal saline. They were diluted in 1 mL of sterile saline solution. Then, 100 µL was added to the soybean–casein digest agar plate with 3% sodium chloride and incubated at 37 °C for 48 h. Bacterial colonies were counted, and the percentage of inhibition was calculated according to the following equation:

Percent of inhibition (%) = ((C−B) ÷ C) ×100, where B represents the bacterial count of the sample, and C represents the bacterial count of the control.

#### 2.9.6. Histological evaluation

Histological evaluation was carried out according to Shu et al. [38]. Briefly, rats were sacrificed on days 3, 5, and 7, and the tissues surrounding the wound were sliced. They were preserved for 48 h in 10% formalin and then dried using different concentrations of anhydrous ethanol. Afterward, samples were embedded in paraffin and brought into a clear state. Slices were sliced to a thickness of 4–7 µm. All sections were stained with hematoxylin and eosin (H&E) and Masson trichrome stain for histological analysis.

### 2.10. Statistical analysis

Correlations between QS genes and biofilm formation, twitching motility, hemolysin production, and antimicrobial resistance were statistically determined using the Chi-square test and the IBM SPSS software package version 25.0. The significance of the results was adjusted at the 0.05 level.

## 3. Results

Based on their appearance on MacConkey agar plates and morphological and biochemical characteristics, 32 (43%) of the 75 clinical isolates were initially identified as *Pseudomonas* spp. Gram staining of non-lactose fermenting colonies on MacConkey agar plates revealed Gram-negative rods. All isolates were positive for oxidase and catalase, negative for indole, methyl red, and Voges-Proskauer tests, and positive for citrate. All isolates produced red butt and slant with no gas or H_2_S on triple sugar iron agar slants. All isolates grew on cetrimide agar plates showing greenish pigment. Using the automated VITEK 2 system, the 25 isolates were identified at the species level. Eleven (44%) *P. aeruginosa* isolates were obtained from blood samples, four (16%) from burn wounds, four (16%) from ascetic fluid, and six (24%) from urine. According to antibiotic resistance, the 25 *P. aeruginosa* isolates were classified into six resistance patterns from R1 to R6 (Table 2). Eighteen (72%) of the 25 *P. aeruginosa* isolates were multidrug-resistant (MDR), and 7 (28%) were extensively-drug resistant (XDR). An MDR isolate is considered to be non-susceptible to at least one agent in ≥3 antimicrobial categories, and an XDR isolate is considered to be non-susceptible to at least one agent in ≤2 categories [39]. The highest resistance (96%) was observed against cefepime, while 56% of the isolates were resistant to meropenem, which was the most effective antibiotic in this study (Table 3).

**Table 2.** Distribution of antibiotic resistance patterns among the 25 *P. aeruginosa* isolates.

Resistance pattern	Number of resistant antibiotics	Number of resistant isolates
R1	10	9
R2	9	3
R3	8	4
R4	7	1
R5	5	2
R6	Less than 5	6

**Table 3.** Antibiotic susceptibility of the 25 *P. aeruginosa* clinical isolates.

Sensitive		Resistant		Antibiotic
%	No.	%	No.	
32	8	67	17	Ceftazidime (CAZ 30 µg)
4	1	96	24	Cefepime (FEP 30 µg)
12	3	84	21	Piperacillin (PI 100 µg)
36	9	64	16	Piperacillin/tazobactam (PIT 100/10 µg)
24	6	76	19	Aztreonam (AT 30 µg)
32	8	68	17	Gentamicin (GEN 10 µg)
36	9	64	16	Levofloxacin (LE 10 µg)
44	11	56	14	Meropenem (MRP 10 µg)
40	10	60	15	Imipenem (IPM 10 µg)
32	8	67	17	Gatifloxacin (GAT 5 µg)

This study investigated several QS-dependent virulence factors, including hemolysin production, twitching motility, and biofilm formation. Among the 25 *P. aeruginosa* isolates, 20 (80%) exhibited twitching motility, and 23 (92%) showed hemolysin production. Additionally, 20 (80%) of the isolates were positive for biofilm formation as determined by both the Congo red agar and microtiter plate methods. Of these biofilm-forming isolates, 8 (40%) were classified as strong biofilm producers, 7 (35%) as moderate, and 5 (25%) as weak.

Using the conventional PCR technique, 23 (92%) of the 25 *P. aeruginosa* isolates were positive for the *lasI* gene, and 24 (96%) were positive for the *lasR* gene.

To investigate the antibacterial effect of the antidepressant drugs (fluoxetine and paroxetine) and levofloxacin on QS-dependent virulence factors, MICs of these drugs were determined against our 25 *P. aeruginosa* isolates using the broth microdilution method. MICs of fluoxetine and paroxetine ranged from 32 to 512 µg/mL (Table 4). Both fluoxetine and paroxetine presented antibacterial activity against *P. aeruginosa* isolates and fluoxetine showed higher antibacterial activity than paroxetine. On the other hand, MICs of levofloxacin ranged from 0.5 to 256 µg/mL against our isolates (Table 4). Half the MICs of fluoxetine, paroxetine, and levofloxacin were selected to investigate the interaction between the fluoxetine/levofloxacin and paroxetine/levofloxacin drugs by FICI and MICs determination. A synergistic outcome was observed in both combinations, and the antibacterial effect of levofloxacin was found to be enhanced (Table 4). A statistically significant correlation was observed between QS genes and biofilm formation, twitching motility, and hemolysin production. However, no significant correlation was found between QS genes and the antimicrobial resistance of the isolates.

**Table 4.** MICs of fluoxetine, paroxetine, levofloxacin, and their combinations with FICI values of combinations against *P. aeruginosa* clinical isolates.

Isolate code	MIC of FX	MIC of PX	MIC of LEV	MIC/2 of LEV + MIC/2 FX	FICI of LEV + FX	MIC/2 of LEV + MIC/2 PX	FICI of LEV + PX
P300	128	256	1	32	32.25	64	64.25
P150	128	512	16	128	9	128	9
P37	128	512	32	16	2.13	16	0.50
P102	512	512	256	32	0.13	256	1.5
P121	512	512	64	128	2.25	64	1.13
P64	128	128	1	64	64.5	64	64.5
P97	128	128	64	128	3	128	3
P301	128	128	256	256	3	256	3
P41	512	128	256	512	3	256	3
P130	512	512	256	16	0.09	16	1.13
P145	128	32	1	64	64.5	64	66
P36	128	128	64	256	6	256	6
P10	128	128	2	256	130	64	32.5
P104	128	128	256	128	1.45	256	3
P99	128	128	1	128	129	32	32.25
P163	64	256	0.5	128	258	128	257
P95	64	128	256	256	5	256	3
302	128	128	1	256	258	256	258
304	128	256	1	256	258	256	257
P2	512	512	64	32	0.56	32	0.56
107	128	128	64	64	1.5	16	0.36
96	32	128	1	256	264	256	258
P1	512	128	1	128	128.25	256	258
P13	512	128	64	128	2.25	128	3
108	512	256	256	128	0.75	256	2

FX: fluoxetine; PX: paroxetine; LEV: levofloxacin; FICI: fraction inhibitory concentration index.

The biofilm inhibition assay evaluated the effects of paroxetine and fluoxetine at half their MICs. Biofilm formation was reduced in 68.18% of isolates treated with paroxetine and 81.18% of those treated with fluoxetine. Besides, the inhibitory effect of paroxetine and fluoxetine at half their MICs on twitching motility was investigated. It was found that no haze of growth was observed at the interface between the plate and the agar surface, which indicates the inhibition of twitching motility by paroxetine and fluoxetine in 35% and 56% of isolates, respectively. Furthermore, the inhibitory effects of paroxetine and fluoxetine at half their MICs on hemolytic activity were investigated. The inhibition of hemolytic activity was achieved by paroxetine and fluoxetine in 88% and 90% of our isolates, respectively.

**Figure 1.**
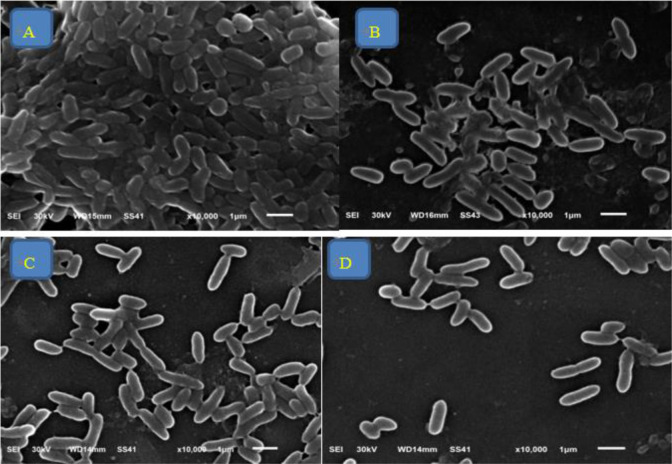
SEM examination of the inhibitory effect of paroxetine, fluoxetine, and levofloxacin on biofilm formation by the *P. aeruginosa* isolate (P1) after 24 h of culture (×10,000 magnification). A. Dense aggregation of *P. aeruginosa* cells with secreted matrices forming the biofilm. B. Effect of paroxetine on biofilm formation. C. Effect of fluoxetine on biofilm formation. D. Effect of levofloxacin on biofilm formation.

Regarding the inhibitory effect of fluoxetine/levofloxacin and paroxetine/levofloxacin combinations (1/2 MIC of each drug), twitching motility, hemolysin production, and biofilm formation were inhibited in all isolates (100%), indicating a synergistic outcome of these combinations. Furthermore, SEM was used to investigate the inhibitory effect of paroxetine and fluoxetine on biofilm formation in *P. aeruginosa* isolate (P1), which was used as a control showing strong biofilm formation. Half the MICs of paroxetine, fluoxetine, and levofloxacin were added to P1 individually. Following 24 h cultivation, *P. aeruginosa* (P1) was found to grow extensively to form an early biofilm (Figure 1A). Following the addition of 1/2 MIC of paroxetine and fluoxetine, bacterial density was significantly reduced, and the sizes of some bacterial colonies diminished (Figure 1B, C). SEM demonstrated a reduced and dispersed distribution of bacterial cells after the addition of levofloxacin to the biofilm at half its MIC (Figure 1D).

**Figure 2.**
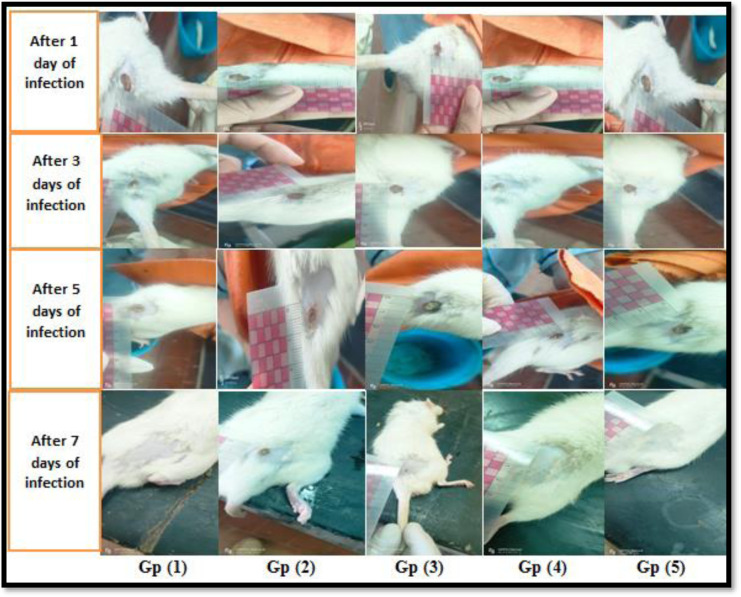
Examination of wound healing and scar formation in rats in five different treated groups over seven days of infection with a selected *P. aeruginosa* isolate. Gp1: wounds without infection or treatment; Gp2: infected wounds without treatment; Gp3: infected wounds with fluoxetine treatment; Gp4: infected wounds with levofloxacin/fluoxetine combination treatment; and Gp5: wound infected with levofloxacin treatment.

The effect of fluoxetine on healing wounds infected by *P. aeruginosa* was investigated in each group by photographing by a digital camera at various intervals (0, 1, 3, 5, and 7 days). The rate of wound closure matched the change in the wound's shape. Wound closure rates in rats treated with fluoxetine and levofloxacin increased significantly over time; however, the group treated with the fluoxetine/levofloxacin combination demonstrated the highest wound-healing effect. On the first day after inoculation of the bacterial suspension, despite the darkness of wounds in all groups, wounds treated with levofloxacin, fluoxetine, and their combination showed relatively lighter color, with the latter being the lightest. On the third day, the wound areas in the groups receiving treatments substantially decreased; however, the scab layer of the control group became thicker and brown showing more inflammation and delayed wound healing. On the fifth day, the effectiveness of the fluoxetine, levofloxacin, and their combination was more apparent during wound healing, showing clear area contraction, compared to the control group. On day 7, wounds treated with fluoxetine, levofloxacin, or their combination were nearly closed, with additional hair growth observed, in contrast to the CMS control group, which exhibited slower wound healing (Figure 2).

Furthermore, the effect of fluoxetine on healing wounds infected by *P. aeruginosa* was investigated *in vivo* on male Wister albino rats by determining bacterial count changes. On the last day of the study, it was obvious that the groups treated with fluoxetine, levofloxacin, or their combination showed an apparent inhibition in bacterial growth compared to CMS (control). The percentages of decrease in bacterial count were 91.27% for levofloxacin, 84.49% for fluoxetine, 95.60% for levofloxacin/fluoxetine combination, and 15.68 % for CMS (Table 5).

**Table 5.** Percentage of inhibition of *P. aeruginosa* growth in rats with infected wounds treated with fluoxetine, levofloxacin, and their combination.

	Percentage of inhibition (mean ± SE)			

Days	Control group (CMS)	Fluoxetine-treated group	Fluoxetine/levofloxacin-treated group	Levofloxacin-treated group
Day 1	35.50 ± 0.92	40.35 ± 1.05	51.13 ± 1.74	49.89 ± 1.74
Day 2	33.54 ± 2.05	55.77 ± 2.47	72.56 ± 0.93	70.25 ± 0.93
Day 3	27.99 ± 2.44	63.97 ± 0.99	81.79 ± 0.25	78.27 ± 0.25
Day 4	21.84 ± 1.57	72.77 ± 0.24	87.91 ± 0.19	84.52 ± 0.19
Day 5	18.50 ± 1.25	80.08 ± 0.62	91.26 ± 0.21	88.64 ± 0.94
Day 6	16.35 ± 2.20	82.26 ± 1.52	93.14 ± 1.06	90.46 ± 1.05
Day 7	15.68 ± 1.76	84.49 ± 1.19	95.60 ± 0.87	91.27 ± 0.78

**Figure 3.**
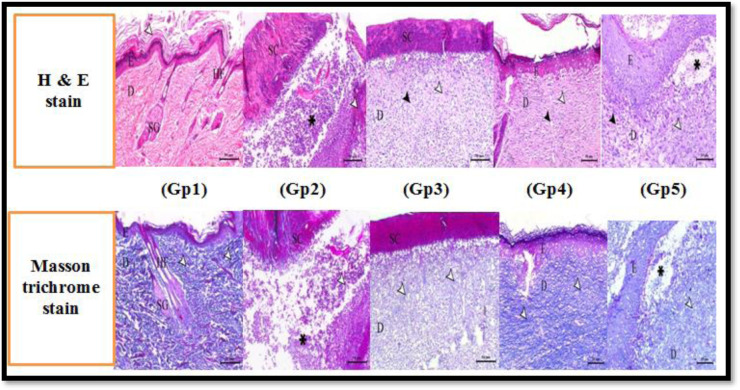
Histological examination of wound healing by H&E stain and Masson trichrome staining in five rat groups with *P. aeruginosa*-infected wounds. In both stains, skin sections of group 1 (control group) showed normal skin layers. Epidermis (E) consisted of stratified squamous keratinized epithelium (arrowheads indicate keratin layer), and dermis (D) consisted of connective tissue containing skin appendages including hair follicles (HF) and sebaceous glands (SG); scale bar = 50 µm. Skin sections of group 2 (infected with *P. aeruginosa*) showed extensive purulent and necrotic changes extending deep into the dermis (arrowheads indicate necrosis and asterisk indicates pus) covered by loose scab (SC). Skin sections of group 3 (infected with *P. aeruginosa* and treated with fluoxetine) showed immature granulation tissue within the dermis (D) associated with mild collagen formation (white arrowheads) and perpendicular blood capillaries (black arrowheads) covered with adherent scab (SC) and with scanty epithelization. Skin sections of group 4 (infected with *P. aeruginosa* and treated with fluoxetine/levofloxacin combination) showed marked epithelization (E) with obvious maturation of the granulation tissue of the dermis (D) associated with remodeling of the connective tissues (white arrowheads) with perpendicular blood capillaries (black arrowheads). Skin sections of group 5 (infected with *P. aeruginosa* and treated with levofloxacin) showed marked epithelization (E) with immature granulation tissue of the dermis (D) associated with marked angiogenesis (black arrowheads), edema (asterisk), and soft collagenous matrix (white arrowheads).

For histological evaluation, rats were sacrificed on days 3, 5, and 7, and sections were stained with H&E stain and Masson trichrome stain using a digital camera (Figure 3). The control group's skin sections clearly showed normal histological components, including intact subcutaneous tissue, dermis, and epidermis. The dermal layer also included a large number of active hair follicles and negligible infiltrates of inflammatory cells. On the other hand, the infected untreated group displayed a significant quantity of granulation tissue along with the diffusion of inflammatory cell infiltrates, with severe epidermal necrosis and sloughing. In addition, there were a few dispersed activated hair follicles and very few collagen fibers. The combination treatment group displayed the fastest rate of wound healing and complete re-epithelialization linked to a somewhat thicker scab that included necrotic debris and large aggregations of inflammatory cells. Furthermore, a larger number of activated hair follicles and moderate inflammatory cell infiltrates were also observed in the dermal layer along with a greater amount of collagen fibers. Mild subcutaneous blood vessel congestion was clearly seen. Moreover, the levofloxacin-treated group showed a higher and accelerated wound-healing process compared with the fluoxetine group.

The deposition of collagen fibers is utilized as an indicator of the healing process; the greater the number of collagen fibers, the more successful the healing method. In this study, the combination-treated group demonstrated a clear significant healing process, with 61.94% collagen fiber deposition, which was comparable to the untreated group's 22.47% collagen fiber deposition (Table 6).

**Table 6.** Percentage of collagen deposition among the five studied groups.

Groups	Characteristics of groups	% of collagen deposition (mean ± SE)
Gp 1	Control group (not infected)	79.02 ± 3.01
Gp 2	Infected with *P. aeruginosa* without treatment	22.47 ± 3.02
Gp 3	Infected with *P. aeruginosa* and treated with fluoxetine	43.35 ± 2.42
Gp 4	Infected with *P. aeruginosa* and treated with fluoxetine/levofloxacin combination.	61.94 ± 2.15
Gp 5	Infected with *P. aeruginosa* and treated with levofloxacin	47.56 ± 2.96

## 4. Discussion

Multidrug-resistant *P. aeruginosa* is a serious health problem since it limits treatment options and lengthens hospital stays [40]. In this study, pan-drug resistant (PDR), XDR, and MDR isolates represented 36%, 24%, and 40% of our *P. aeruginosa* isolates, respectively. The extensive distribution of MDR *P. aeruginosa* isolates matched earlier studies in Egypt [41],[42]. Rodulfo et al. reported higher resistance rates in Venezuela, with the prevalence of MDR and XDR *P. aeruginosa* isolates reaching approximately 71.9% [43]. Besides, Goncalves et al. reported that 73.9% of their isolates were MDR [44]. In contrast, other Egyptian researchers reported a lower prevalence rate of MDR isolates [45],[46]. The high resistance of our isolates may be attributed to their hospital-acquired origin, which is typically associated with elevated resistance levels. It was reported that imipenem resistance rates ranged from 1% to 73.5% [39],[47]–[50]. In this study, 60% of our *P. aeruginosa* isolates were imipenem-resistant, and these isolates also showed significant resistance to other antibiotics including cefepime, piperacillin-tazobactam, and gentamicin. Similar results were reported by Baniya et al. in South Asia and Bogiel et al. in Poland [50],[51]. The excessive usage of carbapenems in the treatment of hospital infections may be the cause of this high incidence of imipenem resistance [46]. It was reported that carbapenem-resistant *P. aeruginosa* often harbored other virulence genes giving rise to an emergent concern [52]. However, other researchers could not uncover any obvious correlations between MDR *P. aeruginosa* virulence genes and antibiotic resistance [53].

*P. aeruginosa* isolates have numerous virulence factors that contribute to their pathogenicity in several diseases. The pathophysiology of *P. aeruginosa* is greatly impacted by virulence factors, and several clinical studies have investigated how *P. aeruginosa* virulence is affected by different factors [54]. In this study, some QS-dependent virulence factors were investigated, namely biofilm formation, hemolysin production, and twitching motility. Regarding twitching motility, 80% of our isolates were positive. In contrast, Deligianni et al. reported that 28% of their *P. aeruginosa* isolates were positive for twitching motility [55]. Similar to our results, Ochoa et al. reported that 75.8% of their isolates were positive for twitching motility [56]. Regarding hemolysin production, 92% of our isolates were positive. Similarly, Aqel et al. in Saudi Arabia reported that 95% of their isolates were positive for hemolysin production [57]. Regarding biofilm formation, 80% of our isolates were positive. Similar to our results, Senturk et al. reported that 78% of their isolates were biofilm producers [58] and several other studies also supported these results [59],[60]. In contrast, Deligianni et al. reported a higher percentage of biofilm production, with all their isolates forming biofilms [55]. In this study, biofilm formation in carbapenem-sensitive and carbapenem-resistant isolates was 70% and 93.44%, respectively. Similar results were reported by other studies [61]. Moreover, other studies reported that biofilm production in MDR *P. aeruginosa* isolates was significantly higher than in non-MDR *P. aeruginosa* isolates [53]. In contrast, other studies have reported no statistically significant correlation between the presence of multidrug resistance and biofilm formation. This may be due to the selective silencing of some genes and the activation of others, or to the decreased virulence of MDR strains, which is controlled by the bacterial genome allowing their survival in the presence of antibiotics [51],[62].

QS plays a vital role in *P. aeruginosa* virulence as it controls several virulence factors. In the present study, we investigated the presence of two QS genes (*lasI* and *lasR*) using the conventional PCR technique. 92% of the 25 *P. aeruginosa* isolates were positive for the *lasI* gene, and 96% were positive for the *lasR* gene. Lower percentages were reported by Ghanem et al.: 80% and 81.6% of their isolates were positive for *lasI* and *lasR* genes, respectively [63]. Besides, Sabharwal et al. reported that 75% of their isolates were positive for both genes [64]. In contrast, Kadhim et al. reported that *lasR* and *lasI* genes were detected in 5% and 78.3% of their isolates, respectively [65]. Similarly, Aghamollaei et al. found that 48.5% of their isolates were positive for *lasI* [66]. According to the results of the present study, the two QS genes (*lasR* and *lasI*) were detected in a high percentage in our highly resistant isolates, indicating that the QS system is crucial for *P. aeruginosa* pathogenicity. Furthermore, the majority of our clinical isolates exhibiting QS genes were classified as MDR.

In order to investigate the effect of the two selected antidepressant drugs (fluoxetine and paroxetine) on QS-dependent virulence factors in our *P. aeruginosa* isolates, their MICs were determined by the broth microdilution method. Both fluoxetine and paroxetine presented antibacterial activity against all our *P. aeruginosa* isolates, with fluoxetine showing higher antibacterial activity than paroxetine. Similar to our results, Sousa et al. reported that fluoxetine showed antibacterial activity against their *P. aeruginosa* isolates with a MIC of 161 µg/mL, which was among our MIC ranges [67]. Half the MICs of fluoxetine, paroxetine, and levofloxacin were selected to investigate the interaction between the fluoxetine/levofloxacin and paroxetine/levofloxacin drugs by FICI and MICs determination. A synergistic outcome was observed in both combinations, with an enhanced antibacterial activity of levofloxacin. MICs of combinations were decreased to a great extent compared with MICs of individual drugs. Similarly, Foletto et al. reported a synergistic effect of ciprofloxacin combinations with either paroxetine or fluoxetine, as evidenced by the reduction in the MICs of the combinations [68]. Moreover, it was reported that SSRIs did not only exhibit antimicrobial activity but also impaired a number of processes incorporating biosynthesis of products in microorganisms such as slime synthesis and swarming. This may indicate their action on basic metabolic processes, which could be either related or unrelated to the uptake of substances [18].

The inhibitory effects of paroxetine and fluoxetine at half their MICs on twitching motility, hemolysin production, and biofilm formation were investigated. In the biofilm inhibition assay, reduced biofilm formation was found in 68.18% and 81.18% of isolates for paroxetine and fluoxetine, respectively. Similar inhibitory results were reported by Guedes et al. and Pereira et al. against *Burkholderia pseudomallei* and *Cryptococcus neoformans*, respectively [69],[70]. Besides, the twitching motility and hemolysis inhibition assays revealed that paroxetine and fluoxetine effectively inhibited both virulence factors in our MDR isolates. Moreover, fluoxetine/levofloxacin and paroxetine/levofloxacin combinations caused inhibition of twitching motility, hemolysin production, and biofilm formation in all isolates (100%), indicating a synergistic outcome of these combinations.

SEM was used to investigate the inhibitory effect of paroxetine and fluoxetine on biofilm formation in a selected *P. aeruginosa* isolate characterized by strong biofilm formation. Bacterial density became sparse, and the size of some bacterial colonies was reduced. Similarly, Pereira et al. reported that both fluoxetine and paroxetine reduced biofilm viability and biomass [70].

In this study, the effect of fluoxetine on healing wounds infected by *P. aeruginosa* was investigated in each group at various intervals by photographing with a digital camera and by determination of bacterial count. The wound closure rates in rats treated with fluoxetine and levofloxacin increased significantly over time; however, the group treated with the fluoxetine/levofloxacin combination exhibited the most pronounced wound-healing effect. On the last day of the study, the groups treated with fluoxetine, levofloxacin, or their combination clearly showed an apparent inhibition in the bacterial growth compared to the control.

Similarly, an improvement in the healing of slow-healing wounds was reported by Kiecka and Szczepanik in Poland [71]. Wound healing is a complicated process that includes tissue remodeling, migration, hemostasis, inflammation, and proliferation [72]. SSRIs may promote wound healing by modulating immune responses and microbiota in the wound bed. Additionally, fibroblasts contribute to the production of collagen, proteoglycans, and glycosaminoglycans, which are key components of the extracellular matrix. Moreover, SSRIs may interact with different inflammatory mediators, which affect wound healing. Furthermore, Farahani et al. revealed that either short-term or long-term fluoxetine treatment increased the skin's ability to repair wounds in rats under chronic stress [73]. In addition, Yuksel et al. [74] suggested that administering short-term paroxetine to rats could accelerate skin wound healing by increasing fibroblast numbers and enhancing epithelialization over time. Additionally, it has been demonstrated that paroxetine slightly increased wound contraction, potentially by inducing myofibroblast contraction or increasing their recruitment to the wound area [75]. Furthermore, it was reported that these drugs have strong antiplatelet and endothelial-protecting properties and it was suggested that SSRI therapy could be taken into consideration as a possible wound treatment method [23].

In this study, the histological evaluation of rats revealed that fluoxetine improved wound healing and re-epithelialization, which included necrotic debris and large aggregations of inflammatory cells. Furthermore, a larger number of activated hair follicles and moderate inflammatory cell infiltrates were also observed in the dermal layer along with a greater amount of collagen fibers. Mild subcutaneous blood vessel congestion was clearly seen. The best results were achieved by the fluoxetine/levofloxacin combination treatment group. In addition, the deposition of collagen fibers is utilized as an indicator of the healing process; the greater number of collagen fibers signifies a more successful healing outcome. In this study, the combination-treated group demonstrated a clear significant healing process, with 61.94% collagen fiber deposition, compared to 22.47% in the untreated group.

It was reported that systemic administration of fluoxetine exhibited anti-inflammatory properties in microglia, splenocytes, and lymphocytes [76]. Moreover, Farahani et al. reported that systemically administered fluoxetine increased the healing of acute surgical wounds in rats that were both psychologically stressed and unstressed; the anxiolytic and analgesic properties of fluoxetine could have contributed to these outcomes [73]. Additionally, it was reported that interleukin-1α, IFN-γ, interleukin-6, interleukin-8, interleukin-12, and other pro-inflammatory cytokines were reduced when SSRIs were used for the treatment of depression [77].

In conclusion, the anti-depressant drugs paroxetine and fluoxetine, when repurposed, have shown considerable antibacterial and biofilm inhibitory effects against MDR clinical *P. aeruginosa* isolates. In addition, fluoxetine showed significant improvement in anti-inflammatory effects and enhancement of wound healing. To our knowledge, this is the first Egyptian study investigating the repurposing of paroxetine and fluoxetine as antibacterial agents. One limitation of the analysis of our results was the low number of isolates studied. Therefore, further studies are needed to understand their applicability as antibacterial agents either as single agents or in combination with other antibiotics.
